# Supplementary material for: The Dilemma of the Level of the Inferior Mesenteric Artery Ligation in the Treatment of Diverticular Disease: A Systematic Review of the Literature
Source: J Clin Med. 2022 Feb 10;11(4):917. doi: 10.3390/jcm11040917 (PMC8880703; doi:10.3390/jcm11040917)
Supplement: Supplementary file 1 [file jcm-11-00917-s001.zip › jcm-1572639-supplementary.pdf]

Supplementary Material S1. Excluded studies.

Study	Reason for the Exclusion
Manigrasso 2021	An observational non comparative study on modified caudal-to-cranial approach to perform laparoscopic left colectomy preserving the inferior mesenteric artery for benign colorectal diseases [1]
Wu 2021	An observational non comparative study on preservation of superior rectal artery in laparoscopically assisted subtotal colectomy with ileorectal anastomosis for slow transit constipation
Peltrini 2020	A video on laparoscopic preservation of the inferior mesenteric artery for diverticular disease [3]
Lauro 2020	A narrative review
Puchkov 2019	In this observational non comparative study, the Authors reported the different type of Inferior Mesenteric Artery but they do not analyse the results [5]
Milone 2017	An observational non comparative study on modified caudal-to-cranial approach to perform laparoscopic left colectomy preserving the inferior mesenteric artery for benign colorectal diseases [6]
Bracale 2013	A mix of patients with benign or malignant pathologies underwent laparoscopic left hemicolectomy with or without inferior mesenteric artery preservation [7]
Anania 2014	The treatment of Inferior Mesenteric Artery is not reported [8]
Patriti 2013	A video on robotic preservation of the inferior mesenteric artery for diverticular disease [9]
Cirocchi 2012	A systematic review and meta-analysis on inferior mesenteric artery ligation during sigmoid colectomy for diverticular disease [10]
Desiderio 2012	A technical note of mesenteric artery preservation for diverticular disease during the treatment of a colovescical fistula [11]
Casciola 2006	An observational non comparative study on laparoscopic colon-sigmoid resection with mesenteric artery preservation for diverticular disease [12]
Trebuchet 2002	The treatment of Inferior Mesenteric Artery is not reported [13]
Ignjatovic 2002	An observational non comparative study on preserving the superior rectal artery in laparoscopic anterior resection for complete rectal prolapse [14]
Messinetti 1998	An observational non comparative study on preservation and peeling of the inferior mesenteric artery for complicated diverticular disease. [15]
Napolitano 1996	An observational non comparative study on preservation and peeling of the inferior mesenteric artery in neoplastic colorectal surgery [16]
Fegiz 1976	An observational non comparative study on preservation of the superior hemorrhoidal artery in resection of the colon and rectum in neoplastic colorectal surgery [17]

References

1 Manigrasso M, Pesce M, Milone M, Anoldo P, D'Amore A, Galasso G, Gennarelli N, Maione F, Vertaldi S, Sarnelli G, De Palma GD. Long-Term Functional Results of a Modified Caudal-to-Cranial Approach in Laparoscopic Segmental Left Colectomy for Diverticular Disease. Gastroenterol. Res. Pract. **2021**, *2021*, 8940682. doi: 10.1155/2021/8940682.

2 Wu CW, Pu TW, Kang JC, Hsiao CW, Chen CY, Hu JM, Lin KH, Lin TC. Preservation of superior rectal artery in laparoscopically assisted subtotal colectomy with ileorectal anastomosis for slow transit constipation. World J. Gastroenterol. **2021**, *27*, 3121–3129. doi: 10.3748/wjg.v27.i22.3121. PMID: 34168413; PMCID: PMC8192293.

3 Peltrini R, Pontecorvi E, Silvestri V, Bartolini C, D'Ambra M, Bracale U, Corcione F. Laparoscopic sigmoid colectomy with preservation of the inferior mesenteric artery for diverticular disease - a video vignette. Colorectal Dis. **2020**, *22*, 1205–1206. doi: 10.1111/codi.15053. Epub 2020 Apr 21. PMID: 32248599.

4 Lauro A, Pozzi E, Vaccari S, Cervellera M, Tonini V. Drains, Germs, or Steel: Multidisciplinary Management of Acute Colonic Diverticulitis. Dig. Dis. Sci. **2020**, *65*, 3463–3476.

5.Puchkov DK, Khubezov DA, Puchkov KV, Semionkin EI., Ogoreltsev A.Yu., Ignatov I.S., Lukanin R.V., Krotkov A.R. Short-term outcomes of laparoscopic elective colonic resections for diverticular disease. Koloproktologia **2019**, *18*, 55–68. https://doi.org/10.33878/2073-7556-2019-18-2-55-62.

6 Milone M, Milone F. Segmental left colectomy: a modified caudal-to-cranial approach. Surg. Endosc. **2017**, *31*, 1487. doi: 10.1007/s00464-016-5100-x. Epub 2016 Jul 21. PMID: 27444827.

7 Bracale U, Lazzara F, Merola G, Andreuccetti J, Barone M, Pignata G. Single access laparoscopic left hemicolectomy with or without inferior mesenteric artery preservation: our preliminary experience. Minerva Chir. **2013**, *68*, 315–320. PMID: 23774097.

8 Anania G, Vedana L, Santini M, Scagliarini L, Giaccari S, Resta G, Cavallesco G. Complications of diverticular disease: surgical laparoscopic treatment. G. Chir. **2014**, *35*, 126–128.

9. Patriti A. Preservation-of-the-Inferior-Mesenteric-Artery-in-Sigmoidectomy-for-Diverticular-Disease. Available online: https://clinicalrobotics.com/preservation-of-the-inferior-mesenteric-artery-in-sigmoidectomy-for-diverticular-disease/ (accessed on 9 September 2021).

10 Cirocchi R, Trastulli S, Farinella E, Desiderio J, Listorti C, Parisi A, Noya G, Boselli C. Is inferior mesenteric artery ligation during sigmoid colectomy for diverticular disease associated with increased anastomotic leakage? A meta-analysis of randomized and non-randomized clinical trials. Colorectal Dis. **2012**, *14*, e521–e529. doi: 10.1111/j.1463-1318.2012.03103.x. PMID: 22632654.

11 Desiderio J, Trastulli S, Listorti C, Milani D, Cerroni M, Cochetti G, Cirocchi R, Boselli C, Parisi A, Mearini E, Noya G. Surgical approach of complicated diverticulitis with colovescical fistula: technical note in a particular condition. Cent. Eur. J. Med. **2012**, *7*, 578–583.

12 Casciola L, Ceccarelli G, Stefanoni M, Spaziani A, Conti D, Bartoli A, Di Zitti L, Valeri R, Bellochi R, Rambotti M. Laparoscopic colon-sigmoid resection with mesenteric artery preservation for diverticular disease. Minerva Chir. **2006**, *61*, 1–8. Italian. PMID: 16568016.

13 Trebuchet G, Lechaux D, Lecalve JL. Laparoscopic left colon resection for diverticular disease. Surg. Endosc. **2002**, *16*, 18–21. doi: 10.1007/s004640090122.

14 Ignjatovic D, Bergamaschi R. Preserving the superior rectal artery in laparoscopic anterior resection for complete rectal prolapse. Acta Chir. Iugosl. **2002**, *49*, 25–26. doi: 10.2298/aci0202025i.

15 Messinetti S, Giacomelli L, Manno A, Finizio R, Fabrizio G, Granai AV, Busicchio P, Lauria V. Preservation and peeling of the inferior mesenteric artery in the anterior resection for complicated diverticular disease. Ann. Ital. Chir. **1998**, *69*, 479–482.

16 Napolitano AM, Napolitano L, Costantini R, Ucchino S, Innocenti P. Skeletization of the inferior mesenteric artery in colorectal surgery. Current considerations. G. Chir. **1996**, *17*, 185–189. Italian. PMID: 8754557

17 Fegiz G, Tonelli F, Rossi P, Di Paola M, De Masi E, Simonetti G. Preservation of the superior hemorrhoidal artery in resection of the colon and rectum. Surg. Gynecol. Obstet. **1976**, *143*, 919–925. PMID: 996711.
